# Depth Perception Based on the Interaction of Binocular Disparity and Motion Parallax Cues in Three-Dimensional Space

**DOI:** 10.3390/s25103171

**Published:** 2025-05-17

**Authors:** Shuai Li, Shufang He, Yuanrui Dong, Caihong Dai, Jinyuan Liu, Yanfei Wang, Hiroaki Shigemasu

**Affiliations:** 1 Division of Optical Metrology, National Institute of Metrology, Beijing 100029, China; lees991119@163.com (S.L.); dongyr@nim.ac.cn (Y.D.); daicaihong@nim.ac.cn (C.D.); liujinyuan@nim.ac.cn (J.L.); wangyf@nim.ac.cn (Y.W.); 2 Academy of Artificial Intelligence, Beijing Institute of Petrochemical Technology, Beijing 102627, China; 3 School of Informatics, Kochi University of Technology, Kami City 782-8502, Kochi, Japan; shigemasu.hiroaki@kochi-tech.ac.jp

**Keywords:** human–computer interaction, virtual reality, human vision, depth perception, binocular disparity, motion parallax, fusion models, 3D space

## Abstract

Background and Objectives: Depth perception of the human visual system in three-dimensional (3D) space plays an important role in human–computer interaction and artificial intelligence (AI) areas. It mainly employs binocular disparity and motion parallax cues. This study aims to systemically summarize the related studies about depth perception specified by these two cues. Materials and Methods: We conducted a literature investigation on related studies and summarized them from aspects like motivations, research trends, mechanisms, and interaction models of depth perception specified by these two cues. Results: Development trends show that depth perception research has gradually evolved from early studies based on a single cue to quantitative studies based on the interaction between these two cues. Mechanisms of these two cues reveal that depth perception specified by the binocular disparity cue is mainly influenced by factors like spatial variation in disparity, viewing distance, the position of visual field (or retinal image) used, and interaction with other cues; whereas that specified by the motion parallax cue is affected by head movement and retinal image motion, interaction with other cues, and the observer’s age. By integrating these two cues, several types of models for depth perception are summarized: the weak fusion (WF) model, the modified weak fusion (MWF) model, the strong fusion (SF) model, and the intrinsic constraint (IC) model. The merits and limitations of each model are analyzed and compared. Conclusions: Based on this review, a clear picture of the study on depth perception specified by binocular disparity and motion parallax cues can be seen. Open research challenges and future directions are presented. In the future, it is necessary to explore methods for easier manipulating of depth cue signals in stereoscopic images and adopting deep learning-related methods to construct models and predict depths, to meet the increasing demand of human–computer interaction in complex 3D scenarios.

## 1. Introduction

With the rapid development of artificial intelligence (AI) technology and the increasing demand for human–computer interaction, stereoscopic display technology represented by virtual reality (VR) and augmented reality (AR) devices is booming all over the world, and has been widely used in medical treatment, education, industry, entertainment, and other fields. However, due to the complexity of AI and human–computer interaction demands, how to enhance the realism is one of the difficult problems for current technology development of stereoscopic displays.

**Figure 1:**
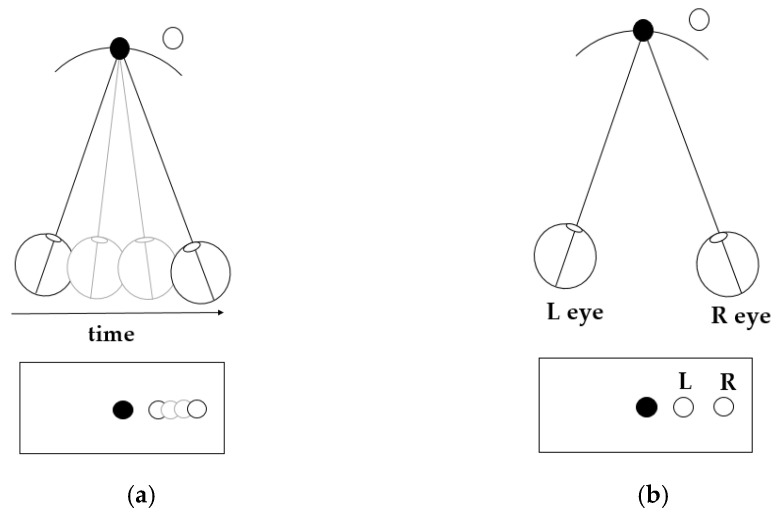
(**a**) Schematic of depth perception generated by motion parallax; (**b**) schematic of depth perception generated by binocular disparity [3].

In natural scenes, the human visual system mainly uses cues such as binocular disparity, motion parallax, shading, and texture to perceive depth in 3D space [1]. Among these, the first two cues are the most commonly used [2,3,4,5]. Binocular disparity is caused by the fact that the two eyes are separated in a certain distance in the horizontal direction. When viewing an object in 3D space, the retinal images in the left and right eyes have small differences, and these small differences will be further processed by the brain to form a 3D depth perception [2]. While motion parallax is caused by the ability that the brain can estimate the depth (or distance) of an object in 3D space based on the movement speed of its retinal image [6], it is a monocular cue, and can be generated by the movements of the objects or the observer themselves. In certain conditions, binocular disparity and motion parallax can produce equivalent depth perceptions [7]. For example, as shown in the top area of Figure 1a, when a monocular observer fixates on the black circle and moves an eye-distance to the right by self-motion, the retinal image shift of the observed object (the white circle in the top area of Figure 1a) generated by this motion parallax cue can be expressed as the distance between the leftmost and rightmost white circles in the bottom box of Figure 1a. While a binocular observer fixates on the black circle (in the top area of Figure 1b) and views the object (the white circle in the top area of Figure 1b) with two eyes simultaneously in a static condition, the difference between the left and right retinal images generated by this binocular disparity cue can be expressed as the distance between the two white circles in the bottom box of Figure 1b. The retinal image shift in Figure 1a is the same as the difference between the left and right retinal images in Figure 1b, thus causing equal depth perception [3].

At present, most stereoscopic display devices are designed based on the principle of binocular disparity. By presenting image signals to the left and right eyes with a certain disparity, depth can be perceived after the observer’s binocular fusion [8]. However, because of the accommodation-vergence (A-V) conflict and extensive binocular disparity caused by this kind of device, there is always visual fatigue which prevents the widespread use of stereoscopic display [9,10,11]. For example, Guo et al. have designed the Go/NoGo paradigm based on different disparity settings and clarified the neural mechanism related to depth perception and stereoscopic visual fatigue in VR [11]. To reduce visual fatigue, some researchers tried to perform nonlinear disparity mapping to compress the binocular disparity in a certain range for stereoscopic display images [12]. However, there are always binocular disparity and motion parallax cues in these images. The nonlinear mapping might induce distortion, overestimation, or underestimation of the perceived depth. Moreover, the constraints of VR devices might also limit the realism of depth reproduction in many scenarios, like the low angular resolution of 3D display which might induce a small range of depth reproduction [13]. Since motion parallax is a depth cue that can be reproduced even in a 2D screen without any limits, some researchers have tried to manipulate both binocular disparity and motion parallax cues to improve the overall realism of depth reproduction in recent years [13].

Thus, it can be seen that to improve the visual comfort and realistic experience of stereoscopic display devices, the premise and key point is the study of depth perception based on the interaction of binocular disparity and motion parallax cues in 3D space. As a result, this review mainly analyzes the mechanisms and interactions of binocular disparity and motion parallax cues on depth perception. Firstly, we introduce the research trends of depth perception and the mechanisms of these two cues; then, we present several depth perception models based on the interaction of these two cues, and compare their respective merits and limitations; finally, we analyze the open challenges and look into the future directions about depth perception study.

## 2. Research Trends of Depth Perception

Regarding the studies on depth perception, in early days, research was mainly based on a single cue such as binocular disparity or motion parallax; later, as research deepened, scientists conducted qualitative studies on the interaction between two cues on depth perception; recently, some quantitative models were constructed for simple dynamic scenarios.

(1) Studies on a single cue of binocular disparity or motion parallax. Early studies on depth perception mainly focused on a single cue. Since Julesz proposed using random-dot stereograms to create binocular disparity images, scientists had conducted depth perception study in 3D space using psychophysical and neurobiological methods [14]. For example, Tyler studied 3D depth perception by using binocular disparity [15], and Rogers and Graham conducted related research using motion parallax cues [16]. Fang’s team, through psychophysical and functional magnetic resonance imaging (fMRI) techniques, identified specific visual areas in the brain that respond to binocular disparity [17]. Other researchers have also explored depth perception study from other cues like color [18,19], but they mainly focused on a single cue, without studying the interaction between these two cues.

(2) Qualitative studies on the interaction of two cues on depth perception. Previous studies reported that there was interaction between these two cues on depth perception [20]. For example, through a series of psychophysical experiments, Bradshaw et al. found that the absolute values of depth perception thresholds obtained based on binocular disparity and motion parallax cues are different, but their depth perception thresholds are very similar when relative to the spatial frequency distributions of the sinusoidal stimuli, indicating that there is a close connection between the two cues on depth perception [21]. After adapting to the same or a different cue of binocular disparity or motion parallax cues, Bradshaw and Rogers found there was a within- and between-cue threshold elevation for 3D structure detection defined by either cue; for a compound stimulus containing both cues, the depth detection threshold was lower than the thresholds defined by either cue separately (namely, there was a sub-threshold summation) [22]. These experiments suggest that there might be a nonlinear interaction between binocular disparity and motion parallax cues [22].

**Figure 2:**
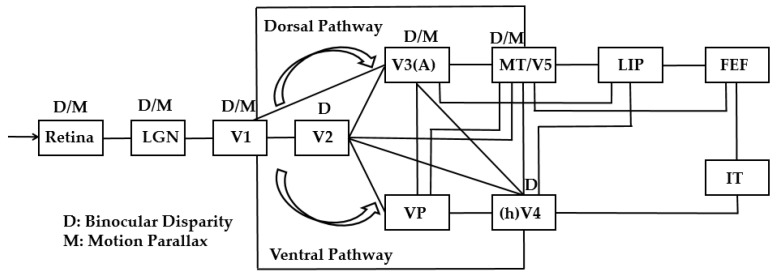
Schematic of visual processing in the visual cortex for binocular disparity and motion parallax cues.

Furthermore, researchers asked macaques or human observers to observe motion parallax- or binocular disparity-specified stimuli, and simultaneously detected their brain activities by using a neurophysiological method or functional nuclear magnetic resonance (fMRI) approach. They found the following: (a) the primary visual cortex (V1, V2, V3, etc.), ventrolateral area (hV4, etc.), and dorsal visual areas (V3A, MT, etc.) show responses to binocular disparity signals [23]. (b) More than half of the neurons in the MT visual area respond strongly to both binocular disparity and motion parallax signals, as evidence of interaction between these two cues [22,24,25,26,27,28]. The schematic of visual processing in the visual cortex for binocular disparity and motion parallax cues can be summarized as shown in Figure 2 [22,24,25,26,27,28,29]. However, these studies mainly focused on qualitative analysis and did not explore the quantitative relationship between these two cues on depth perception.

(3) Quantitative studies with these two cues on depth perception. Qian explained the mechanism of binocular disparity-specified depth perception from the aspect of the neuronal receptive field, and constructed a binocular disparity calculation model based on complex cell response characteristics [30]. Nawrot and Stroyan proposed the “motion/pursuit ratio” law, linking binocular disparity and motion parallax cues to the ratio of depth to viewing distance [31]. In recent years, some researchers have constructed depth perception models, including the WF model, the MWF model, the SF model, and the IC model, to quantitatively analyze the contributions of binocular disparity and motion parallax cues on depth perception [22,32,33,34]. With these models, they revealed that the relationship between these two cues for depth perception might be linear, non-linear, and even more complicated based on different conditions. We will explain these models with more details in Section 5.

## 3. The Mechanism of Binocular Disparity Cue

**Figure 3:**
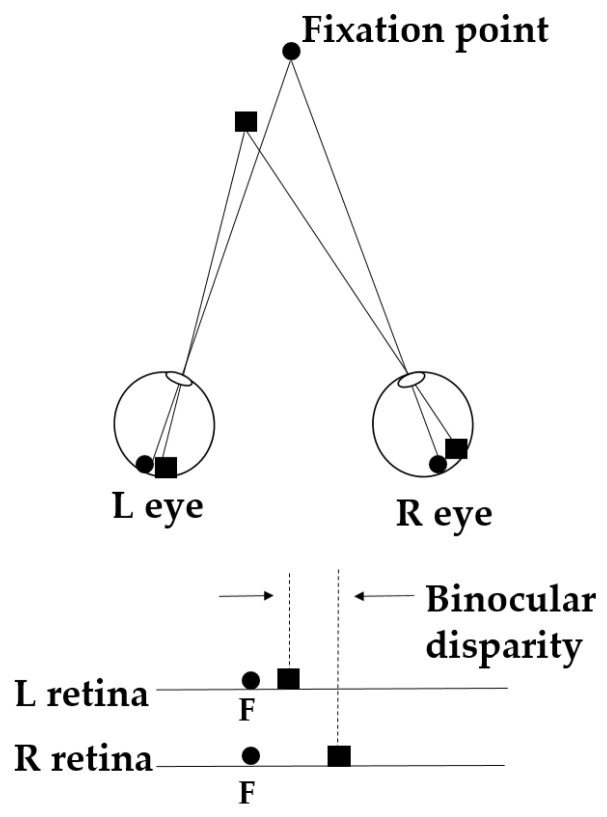
Schematic of the binocular disparity principle [30].

The principle of binocular disparity can be illustrated as the schematic in Figure 3. The upper part of Figure 3 demonstrates how the fixation point (black circle) and the observed object (black square) are projected onto the retinas of the left and right eyes, respectively. Because the distance and viewing angle of the fixation point relative to both eyes are the same, its image is in the fovea of each retina (i.e., point F in the lower part of Figure 3). In contrast, the observed object is located at different distances and viewing angles relative to each eye, resulting in a positional offset between its retinal images in the left and right eyes (the distance between the two dashed lines in the lower part of Figure 3). This positional difference is known as binocular disparity [30].

Binocular disparity used as a cue for depth perception started as early as the 19th century. In 1838, Wheatstone invented the stereoscope and demonstrated the retinal differences between the two eyes could cause stereoscopic vision [35]. Nearly a century later, Julesz used random-dot stereogram stimuli, and found that the brain could only use binocular disparity information to perceive depth, even when other depth cues were absent [14].

Recent research has revealed that the magnitude of binocular disparity can be influenced by spatial variation in disparity. For example, Hibbard explored how the spatial variation in disparity and its second-order luminance statistics had an impact on disparity tuning of the energy model. By modeling natural images using a binocular energy model, the author analyzed the neural responses of the model neurons in various disparity conditions. Results showed that model neurons tuned to small disparities responded most strongly, which was more obvious for vertical than for horizontal disparity, and also related to the eccentricity [36]. In addition, viewing distance is another factor that has impact on binocular disparity-specified depth perception. Studies have shown that when observers view a scene at a close distance, binocular disparity serves as an important depth cue for providing precise depth estimates [37,38,39].

Depth perception specified by binocular disparity is also related to the position of visual field (or retinal image) used. Hibbard and Bouzit found that when the stimuli were shown below fixation or the fixation distance was increased, the perceived depth tended to be closer than fixation [40]. This result was also supported by other physiological studies; neurons responding to the lower visual field tend to be more sensitive to crossed disparity, whereas those to the upper visual field are more sensitive to uncrossed disparity [41].

In addition, the interaction between binocular disparity and other cues can further enhance the accuracy of depth perception. For instance, the combination of binocular disparity with texture direction, convexity, and/or color information can improve the accuracy of disparity estimation and depth perception [42,43,44,45,46,47]. There is a relationship between binocular disparity and luminance, meaning that objects that are lighter seem to be closer [48]. From the physiological aspect, this can be explained by the fact that neurons tuned to brighter stimuli are usually more sensitive to nearer distances, whereas neurons tuned to darker stimuli are more sensitive to farther distances [49].

## 4. The Mechanism of Motion Parallax Cue

**Figure 4:**
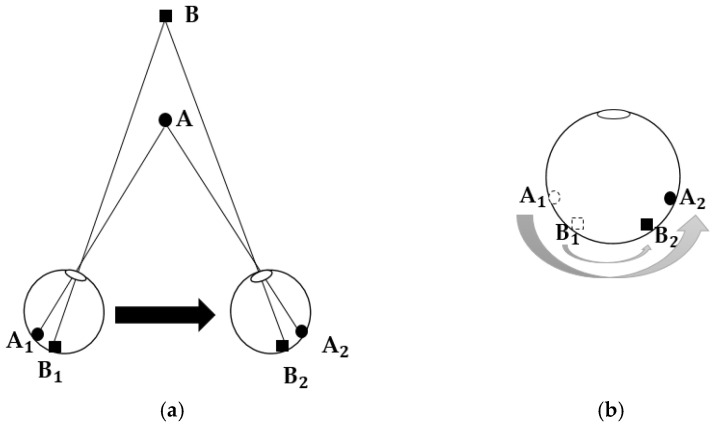
Schematic of the motion parallax principle.

The principle of motion parallax can be illustrated by the schematic in Figure 4. When an observer moves his/her head from side to side and uses one eye (while closing the other) to view objects with different depths in 3D space, the object closer to the observer (object A in Figure 4a) seems to move more on the retina (from A_1_ to A_2_ on the retina) than that (object B, from B_1_ to B_2_) farther away from him/her (as Figure 4b). The human visual system uses the movement speeds of objects on the monocular retina to judge their depths; this is called motion parallax [50].

Motion parallax used as a cue for depth perception also started in the 19th century. With the invention of the stereoscope, Wheatstone also pointed out that head movement could provide equivalent depth perception without the involvement of binocular disparity [35]. In 1925, Helmholtz defined this cue as motion parallax and pointed out that it could produce the same depth perception as binocular disparity [51]. Although some researchers questioned the effectiveness of motion parallax [52,53,54], Rogers and Graham used random-dot stereograms to construct an experimental paradigm of observer- or object-motion, and demonstrated that motion parallax could serve as an independent and effective depth cue [16].

Previous studies reported that head movement and retinal image motion have influence on motion parallax-specified depth perception [55]. Designing different kinds of motion parallax cues, Malla et al. proposed that even slight head movement (e.g., a few millimeters), it will have an influence on depth perception caused by these cues [55]. Fulvio et al. also confirmed that head jitter had an impact on motion-in-depth perception, which was not found in many experiments due to head fixation [56]. In addition, based on the motion parallax cue, Dokka et al. revealed that both observer’s velocity and retinal speed had influence on depth perception [57].

In addition, studies have shown that the combination of motion parallax with other cues may also have an influence on depth perception. Buckthought et al. designed experiments to compare orthographic and perspective rendering, by using textures composed of random-dot and Gabor micro-pattern elements. In these experiments, observers were asked to perform depth sorting tasks in monocular viewing conditions. The results demonstrated that dynamic perspective cues (including small vertical displacement, lateral gradients of speed, and the speed differences between near and far surfaces) can enhance depth perception from motion parallax [58].

Moreover, researchers have also reported the impact of age on motion parallax-specified depth perception. Research by Norman et al. suggested that although older observers may perform less well in 3D shape perception than young people, they can still effectively perceive the magnitudes of depth specified by the motion parallax cue [59]. However, Holmin and Nawrot found that for older adults, their depth thresholds might increase, and the pursuit accuracy might decrease when perceiving depth based on the motion parallax cue. They suggested that these age-related results might be due to the changes of the pursuit signals for the older adults [60].

## 5. Models of Depth Perception Based on Interaction Between Binocular Disparity and Motion Parallax Cues

In practical applications, binocular disparity and motion parallax cues are always coexisting for depth perception. Researchers have revealed that there are interactions between these two cues and their respective contributions to depth perception may vary based on different conditions [38,39,61,62]. This section will introduce several integration models (the WF model, the MWF model, the SF model, and the IC model) with their concepts, advantages/disadvantages, and comparisons.

### 5.1. The WF Model

**Figure 5:**
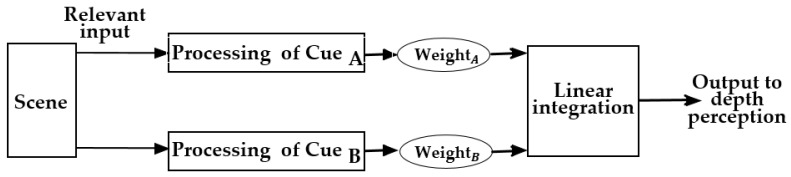
Principle of the WF model [63].

The WF model, also known as the Weak Observer, uses a method of weighted averaging to combine multiple depth cues [32,33]. As illustrated in Figure 5, the main idea of this model is as follows: (1) visual system computes depth maps independently based on each individual depth cue (marked as ${Cue}_{A}$ and ${Cue}_{B}$ in Figure 5); (2) then averages these depth maps to obtain the overall depth for the scene based on linear integration with weights ${Weight}_{A}$ and ${Weight}_{B}$ [34]. Here, take motion parallax and binocular disparity cues as an example; the integration can be expressed as Equation (1).

(1) $$D=\alpha {\cdot D}_{m}+\beta {\cdot D}_{d}$$

where $D_{m}$ and $D_{d}$ represent the depth estimates based on motion parallax and binocular disparity cues, respectively. The weighting coefficients α and β mean the weights of these two cues.

Previous studies used the ancillary measurement method with psychophysical observers to define the weights [64]. In the experiments, researchers used a staircase method to adjust the depth value caused by a single cue until the combined depth was perceived the same as that caused by two constant cues, which can be expressed as Equation (2) [64].

(2) $$D={\alpha \cdot D}_{m}+\beta {\cdot D}_{d}={\alpha \cdot D}_{m}+\beta {\cdot (D}_{m}{+∆}_{cue})$$

where ${∆}_{cue}$ is the amount of the adjusted depth.

Since α+β = 1, they further specialized Equation (2) into Equation (3).

(3) $$\beta =\frac{D-D_{m}}{{∆}_{cue}}$$

With this method, the weight β can be calculated as the ratio of the change in estimated depth to perturbed ${∆}_{cue}$ , which can be obtained via psychophysical experimental data.

The advantages of this model are as follows: it is modular, and the rule of combination (weighted averaging) is simple. Based on this model, there is interaction among different cues only if they share common retinal input. However, since each depth cue might provide different depth information (in a different physical unit), it does not really make sense to make an average of this depth information, which might be a major problem of this model [64].

### 5.2. The MWF Model

Based on the WF model, researchers found that the interaction of binocular disparity and motion parallax is not just a linear combination, which cannot be simply defined as weak fusion [34]. Hence, they proposed an MWF model, which takes consideration of the interaction between cues, and involves two more stages as “cue promotion” and “dynamic weighting”, when comparing with the WF model [34].

**Figure 6:**
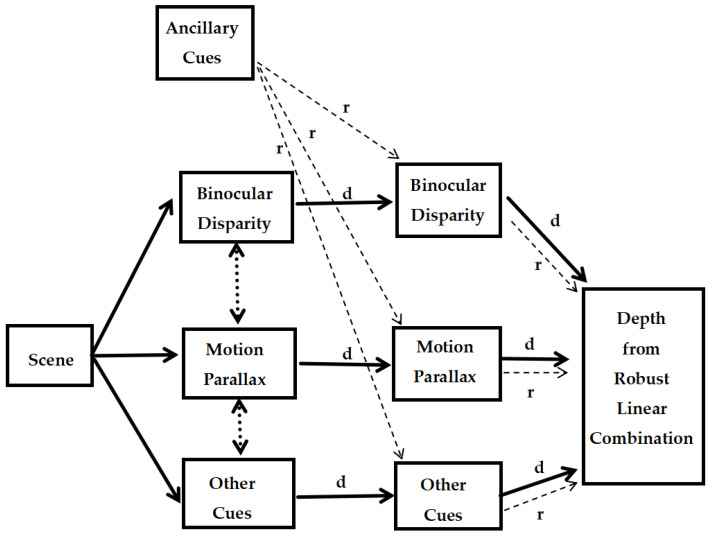
Principle of the MWF model [34].

As shown in Figure 6 (d means depth map, and r means reliability map), the main procedure of the MWF model is as follows:

(1) Cues interact for the purpose of cue promotion, and a depth map is produced for each cue.

Since depth maps estimated from different cues might be in different physical units (e.g., in meters or ratios with dimension 1), there should be a process to change them into common units, which is shown as the interactions between cues (two-way arrows in the left part of Figure 6), namely “cue promotion” [34].

For example, depth from motion parallax might be an absolute measure ( ${depth}_{p}$ ) given by Equation (4) [34]:

(4) $${depth}_{p}=f_{p}(velocity)$$

where ${depth}_{p}$ is the distance from the observer to the object, which is a function of velocity as $f_{p}(velocity)$ .

Depth from binocular disparity ( ${depth}_{s}$ ) can be expressed as Equation (5) [34]:

(5) $${depth}_{s}={d+d^{2}f}_{s}(disparity)$$

where d is the unknown viewing distance, $f_{s}(disparity)$ is the relative depth. After scaling by viewing distance as ${d^{2}f}_{s}(disparity)$ , it is an absolute depth.

To promote the binocular cue, one can use Equation (6) to minimize the inconsistency between the disparity and motion parallax-specified depths and obtain a more stable-estimated—viewing distance d [34].

(6) $$\underset{d}{\min}\sum_{x,y}{\left[{d+d^{2}f}_{s}(disparity;x,y)-f_{p}(velocity;x,y)\right]}^{2}$$

(2) Using ancillary cues, each depth cue produces a depth map and a reliability map. Note that ancillary cues are cues (e.g., vestibular input) which concern the reliability of depth cues when conveying information.

(3) Depth from robust linear combination. Since the reliable depth information from each cue might vary across the scene, thus its weight might change accordingly, this is the process of “dynamic weighting” [34]. If the variance $\sigma_{i}^{2}$ of each cue is known, the weight $\hat{x}$ for each estimated value $x_{i}$ can be defined by its inverse variance, as shown in Equation (7) [34].

(7) $$\hat{x}=\frac{\sum_{i=1}^{n}\frac{x_{i}}{\sigma_{i}^{2}}}{\sum_{i=1}^{n}\frac{1}{\sigma_{i}^{2}}}$$

The weights can also be measured by experiments. For example, researchers constructed two groups of stimuli specified by textural and motion cues for depth perception. The two groups of stimuli were displayed side by side, in which one side was in the consistent-cues condition, the other side in the mixed-cues condition. They asked subjects to indicate which side appeared to have larger depth, and the point of subjective equality (PSE) for each consistent-cues condition can be obtained. Finally, the slope of the PSE distribution suggested the weight of the textural cue was 0.46 [34]. It was found that the lower reliability of a cue, the lower weight it receives [34]. Moreover, the MWF model also can be approximated by using Bayesian methods.

This model has advantages like the involvements of cue interaction and cue modularity, which make better robustness and reliability for depth estimation, and can be used to predict depth across variable scenes [34,65].

### 5.3. The SF Model

**Figure 7:**
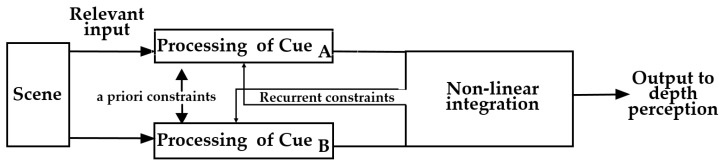
Principle of the SF model [63].

The SF model, also known as the Strong Observer, is an alternative to the WF model [34]. Figure 7 shows how the depth perceived from each cue is integrated in the SF model: the processing of the two depth cues ${Cue}_{A}$ and ${Cue}_{B}$ (for example, binocular disparity and motion parallax cues, respectively) is not independent, but with mutual improvement through a priori constraints and recurrent constraints (namely, a feedback loop). After that, the outputs from these processings are integrated in a nonlinear way to produce output to depth perception [63].

In a priori constraints process, fusion occurs by one cue changing a priori constraints on another cue. For example, in a stereo-vision algorithm, a smoothness constraint is altered to keep the extracted depth smooth. This process can be expressed as an energy minimization procedure as Equation (8) [66].

(8) $$E(\vec{x})=\int {(I}_{l}(\vec{x})+I_{r}{\left(\vec{x}+\vec{D}(\vec{x})\right)}^{2}+(e(\vec{x})-1)\|\nabla \vec{D}(\vec{x}){\|}^{2}d\vec{x}$$

where $\vec{D}(\vec{x})$ represents the disparity field, $I_{l}$ and $I_{r}$ respectively represent the intensity fields of the left and right images. The variable $e(\vec{x})$ functions as the (binary) field of occluding edge locations ( $e(\vec{x})=1$ at an occluding edge, and is zero elsewhere). The occluding edge field is produced from a module that is independent of the disparity field estimation process [66].

In recurrent constraints process, a feedback loop is involved. Since this is a dynamic process, which might cause divergence or oscillation, the convergence and stability should be taken into consideration [66].

Finally, in the non-linear integration process, coupled Markov Random Field (MRF) method, Bayesian formulation, and other methods can be used for data fusion [66].

Unlike the WF model, the SF model does not estimate depth based on the information from different cues in modular, but more likely from the retinal data; thus, the procedure is not modular [34,67].

For example, Ichikawa et al. studied whether and/or how the integration of multiple cues for depth perception at near-threshold levels depended on cue types or the consistency of depth information from each cue [63]. In their experiments, they used sinusoidal stimuli with three depth cues (binocular disparity, motion parallax, and monocular configuration). The sinusoidal stimuli were manipulated in different spatial frequencies and different phases. Experimental results show, in binocular disparity and motion parallax cue condition, when cues specified the same spatial frequency and phase (in-phase condition), these two cues were integrated in the strong fusion process; while if cues specified different spatial frequencies or different phases (out-of-phase condition), the integration was a weak fusion [63]. In addition, in binocular disparity and monocular configuration cue condition, the integration was also a weak fusion process [63]. It can be seen that the visual system integrates depth cues differently depending on the cue types and their consistency. This suggests that cue integration is hierarchical and context-dependent, with early-stage interactions for disparity and parallax and later-stage integration for monocular cues. These findings highlight the complexity of depth perception [63].

### 5.4. The IC Model

Since there is noise in measurement, the estimated depth from each separate cue might have error. Therefore, to reduce the measurement noise, Domini et al. proposed the IC model, which can obtain a composite depth by pooling all depth-related cues together [22].

**Figure 8:**
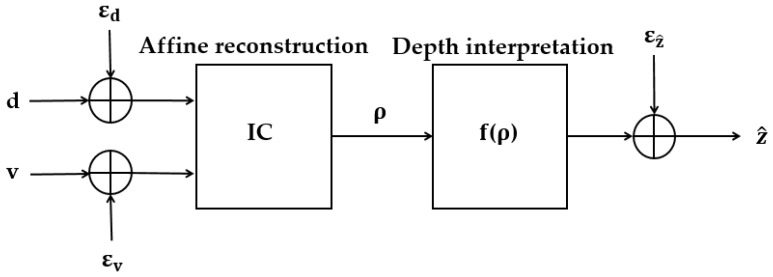
Principle of the IC model [68].

As shown in Figure 8, when processing disparity and velocity signals, the model contains two stages [68].

The first stage is “dimensionality reduction” to obtain the best affine structure estimate. A Principal Component Analysis (PCA) was used to combine disparity signals $\bar{d_{i}}$ and velocity signals $\bar{v_{i}}$ to generate a composite score $\rho_{i}$ which is highly correlated with the scaled depth $z_{i}$ [22]. The main equations are as follows:

(9) $$\bar{d_{i}}\approx \bar{\mu }z_{i}+\varepsilon$$

(10) $$\bar{v_{i}}\approx \bar{\omega }z_{i}+\varepsilon$$

(11) $$\rho_{i}=z_{i}\sqrt{{\bar{\mu }}^{2}+{\bar{\omega }}^{2}}+\varepsilon$$

where $\bar{\mu }$ and $\bar{\omega }$ are the scaled convergence angle and angular velocity, respectively, and ε represents measurement noise. With this method, the best estimate of the affine structure can be obtained based on the first principal component ${PC}_{1}$ .

The second stage is “depth interpretation,” where the model recovers the true Euclidean depth $z_{i}$ from the composite score $\rho_{i}$ through a maximum likelihood estimation method [22]. The main equations are as follows:

(12) $${\hat{z}}_{i}=argmax_{z_{i}}p(\rho_{i}|z_{i})$$

(13) $$p(\rho |z_{i})\propto \int_{R}p\left(\rho |z_{i},R\right)p\left(R\right)dR$$

Here, R represents the global rotation, $p\left(\rho_{i}|z_{i},R\right)$ is the probability of the score given depth and rotation, and $p\left(R\right)$ is the prior distribution of rotation. Since the specific prior distribution is usually unknown, the model assumes that the perceptual outcome primarily depends on the composite score $\rho_{i}$ . With this model, the visual system can obtain the most stable interpretation to the perceived depth [22].

### 5.5. Comparisons and Applications of Above Models

#### 5.5.1. Comparisons of Above Models

To validate the WF, MWF, and SF models, Fine and Jacobs used ellipses with variable widths and depths as stimuli, and positioned them at different viewing distances [34,69]. The stimuli were set with three kinds of information: disparity, retinal motion, and vergence angle; and also in three noise conditions. The tasks were to perform depth judgement (the distance from the nearest point to the farthest point of the ellipse) or shape judgement (the ratio of the depth to the width of the ellipse). They compared the predictions of these three models with the performance of human observers [62]. Experimental results show the following: (i) In the no-noise condition, each model performed well on both tasks. While after adding noise, the shape judgement task seemed to be easier than the depth judgement task. The MWF model performed best in the depth judgement task, and was equivalent to the SF model in the shape judgement task, while the weak fusion model performs poorly. (ii) In the MWF model, the weights of motion and disparity cues were affected by factors like task, viewing distance, and noise model. In the shape judgement task, the weight of the motion cue was higher; while in the depth judgement task, the weight of the disparity cue was higher, and with the increase in viewing distance, the weight of the disparity cue increased. (iii) However, in some conditions or after adding noise, the prediction of the MWF model was inconsistent with the psychophysical data. For example, the weight of the disparity cue in the MWF model increased at a long distance, while in the psychophysical study, human observers relied more on motion cues [34,69].

Tassinari and Domini implemented an experiment to compare the IC/MWF predictions with human observers’ performance [34,69]. In their experiment, they adopted a haploscope with two CRT monitors to present an elliptical hemicylinder specified by random-dot stereograms as stimuli. The elliptical hemicylinder was elongated in one of five different values, and presented in stereo-only, motion-only, and stereo-motion conditions [34,69]. The observers’ task was to judge whether the hemicylinder had larger or smaller depth than an apparently circular cylinder (ACC). Experimental results showed that in the MWF model, although the observer’s point of subjective equality (PSE) in the stereo-only condition was closer to the veridical value than that in the motion-only condition, the PSE in the stereo-motion condition was closer to the value in the motion-only condition; the predicted depth of the MWF model in the stereo-motion condition was close to the observer’s PSE in the stereo-only condition. These results suggest that the predictions of the MWF model could not totally match with observers’ performance [34,69]. They discussed that this might be due to two reasons: it is still unclear how to guarantee a veridical Euclidean solution for each separate depth cue in the cue promotion stage; it is unspecified how to perform the dynamic cue re-weighting (for example, providing different weights to the same cue, which were influenced by the other cues in the scene) [34,69].

However, for the IC model, taking one observer ABB as an example (similar as most of the other observers in this experiment), the ACC was perceived for a simulated depth as in 18.21 mm in the stereo-motion condition, which was highly consistent with the observer’s psychophysical performance (PSE = 18.02 mm), suggesting the effectiveness of the IC model [34,69]. Recently, Domini proposed that the IC model worked in a vector sum model, which used components of a multi-dimensional vector to represent individual cue estimates, and their norms determined the combined outputs. The IC model could explain not only the findings which were in support of the maximum-likelihood estimation (MLE) model, but also those which contradicted the MLE model [70,71,72].

As a result, comparisons of the WF, MWF, SF, and IC models can be summarized as shown in Table 1. In the no-noise condition, each model performs well. However, after adding noise, compared with the WF and SF models, the MWF model performs best in the depth judgement task; but in some conditions, the prediction of the MWF model is inconsistent with the psychophysical data. The IC model pools all depth cues to obtain the most stable interpretation of depth. It predicts systematic bias in performance, which is consistent with observers’ psychophysical performance and also concerns the variability of their performance, but the underlying mechanisms still need to be clarified [22,68,71,72].

#### 5.5.2. Applications of Above Models

The above models can be used in many fields, like VR/AR, 3D television (TV), and so on. For example, Temel et al. proposed a feedback-based modified weak fusion (MWF) model to reconstruct the depth maps at the receiver side of 3D TV [73]. In this model, from the source side, each monocular cue (like color, motion, texture, and so on) in the depth-free streaming of 3D video was generated and sent; the combination of monocular cues at the receiver side was addressed as a nonlinear optimization problem with linear constraints. The initial weights were obtained by particle swarm optimization based on Peak Signal-to-Noise Ratio (PSNR), and then optimized by Active-Sets based on 3VQM (Video Quality Metric). The experimental results show that the quality of the combined depth maps is equivalent to that of the views based on ground truth depth maps, but the bandwidth savings were as high as 38.8% [73].

Some researchers reported that there was a superadditivity of depth cue combination (namely depth overestimation) for 3D shape estimation in VR. Campagnoli et al. used the IC model to predict the superadditivity, and found the prediction by this model was consistent with human observers’ performance [74].

## 6. Open Research Challenges and Future Direction

### 6.1. Open Research Challenges

(1) How to apply depth perception models to improve the realistic experience of depth perception in 3D space

As summarized in Section 5.5, the above models have different complexity and applications. However, based on our investigation, we did not find many applications of the above models. So how to apply these models to improve the realistic experience of depth perception in virtual environments is very important.

In one aspect, it is necessary to further improve the above models. For example, for the MWF model, we can further improve the noise model and consider the deviation of human observers in estimating viewing distance, so as to make it more consistent with the psychophysical data; for the IC model, we still need to clarify the underlying mechanisms.

In the other aspect, it is necessary to further improve the methods of manipulating the 3D sequence. Kellnhofer et al. conducted psycho-visual experiments to study the influence of motion parallax in stereo 3D display, and built up a joint disparity-parallax computational model [13]. To apply this model in autostereoscopic displays, at first, they extracted the optical flow and 3D transformations from the input stereo 3D sequence; then, they used their model to predict depth specified by motion parallax and binocular disparity cues; after that, they performed sampling and estimated necessary scaling for disparity gradients; finally, they integrated the scaled gradients to construct a new output stereo 3D sequence [13]. Furthermore, they evaluated whether the realistic experience of the stereoscopic content was improved after using their method, and results showed a statistically significant 60% preference of the 3D sequence with their method. However, how to extract the optical flow and 3D transformations, estimate the scaling for disparity gradients, and integrate the scaled gradients to improve the preference is still a challenge.

(2) How to build up depth perception models for applications in complex 3D scenarios

As mentioned before, binocular disparity is a cue in a static scene and plays an important role especially in near-distance distances [61]. In contrast, motion parallax is a dynamic scene cue, important for far-distance depth perception, like object tracking and navigation [38,39]. Each cue has different contribution on depth perception at different viewing distances. Moreover, there are also other cues, like contour occlusions, texture, lightness, shading, and perspective, that might have influence on depth perception. This evidence indicates the challenge of depth perception study since it can be affected by multiple viewing conditions and cues [38,39,75].

Moreover, although Kellnhofer et al. obtained a statistically significant 60% preference of the 3D sequence with their method as described in Section 6.1 (1), they still found a problem: their model was built up based on motion parallax and binocular disparity cues, but they applied this model for complex images, which might limit the performance of depth enhancement [13].

So how to build up depth perception models for applications in complex 3D scenarios is another challenge.

(3) How to improve the performance of human–computer interaction via the study of depth perception in 3D space

As human–computer interaction is commonly applied in virtual environments (like VR, AR), how depth perception in 3D space has influence on human–computer interaction is also important. By asking participants to perform continuous Fitt’s pointing tasks with a computer mouse, Cheng and Lin investigated how depth perception in VR environments had an influence on hand–eye coordination, and found that factors like angle of declination (looking downward 20° or straight-ahead vision), visual depth (stereoscopic viewing or monoscopic viewing), space cue (no space, closed space, or closed space with shadow), and index of difficulty (different sizes and distances) had an influence on the performance of depth perception, thus had an impact on the hand-movement performance [76].

These results indicate that depth perception has an influence on human–computer interaction, and it is also a challenge to improve the performance of human–computer interaction since there are multiple factors that have influence on depth perception.

### 6.2. Future Directions

(1) Exploring methods for easier manipulating depth cue signals in stereoscopic images

Although many researchers have been devoted to studying depth perception models based on interaction of different cues, how to apply these models to improve the quality of stereoscopic images is equally important. Up to now, there are some methods to apply models: some are mainly focusing on enhancing apparent depth, without expanding the overall disparity range; but they might not consider the contribution of the motion parallax cue [77,78]. Others account for both binocular disparity and motion parallax cues, and use disparity mapping method to reallocate disparities [13]. In their methods, they compressed disparity for regions which contain motion parallax information, and used the additional disparity budget in other static regions; but there are still some challenges to be solved as mentioned in Section 6.1 (1) [13].

In the future, it is necessary to explore methods (like optimizing hardware and software designs, improving algorithms) for easier manipulating of depth cue signals in stereoscopic images.

(2) Adopting deep learning methods to construct models with multiple cues and/or factors and predict perceived depth for human visual system in complex 3D scenarios

As mentioned in Section 6.1, to solve the challenging problems of building up models in complex 3D scenarios and improving the performance of human–computer interaction, it is necessary to design experimental paradigms that are more in line with the actual complex 3D scenarios; conduct research on depth perception based on the interaction among multiple cues or factors; and construct depth perception models with high efficiency and robustness based on multiple cues’ (or factors’) interaction.

Similar to the human visual system, depth perception is also very important for many high-level computer vision applications, like object reconstruction, scene understanding, autonomous driving, and so on [79,80]. In the above fields, deep learning methods (including artificial neural network, ANN; convolution neural network, CNN) are widely used to predict 3D depth from monocular- and binocular cues of 3D images [79,80]. For example, by using the depth from focus/defocus (DfF/DfD) and stereo matching as the monocular- and binocular information, Chen et al. emulated human perception and exploited unified learning-based hybrid techniques to combine the DfF/DfD and stereo matching information for depth perception [79]. In their experiments, at first, they constructed a comprehensive focal stack dataset, which contained stereo color images and ground truth disparity maps; then, they produced refocused images at each focal layer by using the algorithm from Virtual DSLR (digital single lens reflex); next, they proposed different network architectures for different inputs (including different combinations of the above monocular and binocular cues), integrated and optimized the separate networks to obtain high-fidelity disparity maps; and finally obtained the estimated depth with significantly improved accuracy and robustness [79]. In gesture recognition studies, Almasre and Al-Nuaim compared four support vector machine (SVM) classifiers with two depth sensors to recognize the hand gestures of Arabic Sign Language (ArSL) words [81].

As a next step, we may also use deep learning-relevant methods to construct models with multiple cues and/or factors and predict perceived depth for the human visual system in complex 3D scenarios.

## 7. Summary

Since AI and human–computer interaction are commonly applied in virtual environments (like VR, AR), how to improve the realistic experience of depth perception in virtual environments is very important. To this end, the premise and key point is the study on how the human visual system perceives depth in 3D space. Hence, this manuscript investigates the studies about depth perception based on the interaction of binocular disparity and motion parallax cues. At first, motivation and development trends of depth perception specified by these two cues are introduced. Then, the mechanisms of these two cues are presented. Binocular disparity-specified depth perception is mainly influenced by factors like spatial variation in disparity, viewing distance, position of the visual field (or retinal image) used, and interaction with other cues; while motion parallax-specified depth perception is mainly influenced by factors such as head movement and retinal image motion, interaction with other cues, and the observer’s age. Next, several models, including the WF model, the MWF model, the SF model, and the IC model for depth perception based on the interaction of these two cues are explained and compared. Based on these investigations and summaries, we provide perspectives of open research challenges regarding how to build up and/or apply depth models in 3D complex scenarios, and how to improve the performance of human–computer interaction via the study of depth perception in 3D space. Finally, the new insights for future directions as exploring methods for easier manipulating of depth cues and adopting deep learning methods to construct models and predict perceived depth are proposed, to meet the increasing demand of human–computer interaction in complex 3D scenarios.

## Figures and Tables

**Table 1:** Comparisons of the WF, MWF, SF, and IC models.

Model	Main Procedure	Characteristics	Validation Results
WF [32,33,34,63,64]	(1) Linear average	(1) Modular	(1) In the no-noise condition, each model performs well. (2) While after adding noise, the WMF model performs best in depth judgement task; but in some conditions, the prediction of the MWF model was inconsistent with the psychophysical data.
		(2) Cue independent (no interaction)	
SF [34,63,66,67]	(1) A priori constraints	(1) Non-modular	
	(2) Recurrent constraints	(2) Unconstrained nonlinear interaction	
MWF [34,65]	(1) Cue promotion	(1) Modular	
	(2) Dynamic weighting	(2) Constrained nonlinear interaction	
IC [22,68,70,72]	(1) Affine reconstruction	(1) Non-modular for cues	The IC model could explain both findings which were in support of the MLE model, or contradicted the MLE model.
	(2) Depth interpretation	(2) Concern of reducing the measurement noise	

## Data Availability

Not applicable.
